# The Prognosis of Croup

**Published:** 1887-01-01

**Authors:** 


**The Prognosis of Croup.**—Dr. F. Forcheimer, professor of physiology in the Medical College of Ohio, physician to the Good Samaritan and Children's Hospitals, Cincinnati, in a recent paper before the Cincinnati Academy of Medicine, discussed in an interesting manner the above caption. Nothing, he thought, was so satisfactory and, perhaps, so profitable to the physician in his daily rounds, as an accurate prophecy concerning the course of a disease. In other words, to tell whether our case will be one of the 26.7 per cent. of cases of croup which recover or not. He did not think that medicinal treatment had very much effect on the diphtheritic membrane after it has extended into the larynx, but usually does more harm than good, especially the administration, with lavish hand, of emetics and caustics.

The next point in treatment which affects prognosis is, shall trachetomy be performed? Out of 12,736 cases of diphtheritic croup collected by Monti which had been tracheotomized, 26.7 per cent. recovered. Admitting that some of these cases were fibrinous croup, yet the disparity is too great. For a good prognosis, the operation should be performed in every case, provided the physician has assured himself that laryngeal stenosis is present and has become dangerous to the patient. Seven of the author's cases were not tracheotomized, and but one recovered. The remaining twenty-five cases were operated upon and six recovered.

He has found the operation of intubation to fulfill all the indications of tracheotomy as far as the laryngeal stenosis is concerned. As yet it is quite too early to decide whether intubation will supplant tracheotomy or not. Cæteris paribus, the patient stands a much better chance with intubation than tracheotomy; there is no anæsthetic, no hemorrhage, no after-effects from septic infection or pneumonia. One or the other or both operations are to be performed in all cases, irrespective of age, sex or condition of the patient. In the cases in which the author had resorted to intubation, five in number, the stenosis was as effectually relieved as by tracheotomy. The lumen of the tube he thought sufficiently large to admit air readily. In one of his cases the tube had been swallowed, from faulty method of securing it.

General considerations have a very important bearing on the prognosis. Age, power of resistance, temperature, intensity, and extensiveness of the affection, must be carefully weighed. There are epidemics in which the mortality is simply frightful, do what we may. The further the child has gone into the stage of asphyxia, the less are the chances for his recovery. Much depends upon ausculation of the lungs. The presence of albumen in large quantities, as much as five per cent., makes the prognosis very grave, especially if there are blood clots found. If at any time after the operation the cough becomes dry or the expectoration ceases, we must fear the worst. A sudden rise of temperature — 103° to 104° F.— is a serious symptom, and usually means a new deposit of membrane; increased frequency of respiration is always suspicious. There are very many complications which may cause death after the removal of the canula. These are local and remote. The former may be divided into diphtheritis of the wound, hemorrhages, abscesses. The latter, general septic troubles, followed by one of the most intense forms of anæmia, myocarditis, endocarditis, paralysis, trouble with the kidneys, and finally with the lymphatics. Prognostication is a very difficult task, yet made easier and in some cases exact by taking into consideration all the factors discussed above.—*Med. and Surg. Reporter.*

